# Association of *XRCC6 -61 C > G* and *XRCC7 6721G > T* polymorphisms with susceptibility to pediatric acute lymphoblastic leukemia

**DOI:** 10.1186/s43046-026-00402-6

**Published:** 2026-10-16

**Authors:** Mani Ramzi, Soodabeh Zare, Nasrin Noshadi, Ghasem Nikfar, Zahed Karimi, Mahdiyar Iravani Saadi

**Affiliations:** 1 Shiraz University of Medical Sciences, Shiraz, Islamic Republic of Iran; 2 Fasa University of Medical Sciences, Fasa, Islamic Republic of Iran

**Keywords:** ALL, *XRCC7 6721G > T*, *XRCC6* -*61C > G*, DNA repair, Polymorphism

## Abstract

**Background:**

Acute lymphoblastic leukemia (ALL) is the most frequent malignancy in children. The XRCC6 and XRCC7 genes play a key role in the nonhomologous end-joining (NHEJ) repair process and may influence susceptibility to ALL. The aim of this study is to evaluate the polymorphisms *XRCC6 -61 C > G (rs2267437)* and *XRCC7 6721G > T (rs7003908)* in children with ALL.

**Materials and methods:**

Genomic DNA was extracted from the buffy coat from whole blood samples of patients. Genotyping of *XRCC6 -61 C > G* and *XRCC7 6721G > T* polymorphisms was performed by PCR followed by restriction fragment length polymorphism (PCR-RFLP) using specific restriction enzymes (*BanI* and *Pvu II*).

**Results:**

In this investigation significant associations were discovered between *XRCC6 -61 C > G* genotypes CC (*P* = 0.006) and CG (*P* = 0.01) with ALL patients. It is important to note that while the CC genotype association remained significant after Bonferroni correction (*P* < 0.01), the CG genotype association was nominal (P between 0.01 and 0.05) and should be interpreted with caution. *XRCC7 6721G > T* genotypes GT (*P* = 0.02) and TT (*P* = 0.05) were significantly associated with ALL in males and females, respectively. In ALL patients with B-Cell subtype, *XRCC6 -61 C > G* genotypes CC (*P* = 0.04), GC (*P* = 0.05), and C allele (*P* = 0.05) showed significant associations. *XRCC7 6721G > T* genotypes GT (*P* = 0.003) and TT (*P* = 0.05) were also significantly associated in B-Cell ALL. Significant associations were observed for *XRCC6 -61 C > G* genotype CC (*P* = 0.04) and C allele (*P* = 0.03) in ALL patients with favorable cytogenetic abnormalities.

**Conclusions:**

In conclusion, our findings suggest that the *XRCC6 -61 C > G* and *XRCC7 6721G > T* polymorphisms may be associated with susceptibility to pediatric ALL, with potential lineage-specific and cytogenetic context-dependent effects. However, given the exploratory nature of our subgroup analyses and the statistical limitations, these results should be interpreted cautiously. Large-scale, multi-center studies with functional validation are needed to confirm these associations and elucidate the underlying mechanisms.

**Graphical Abstract:**

**Figure:**
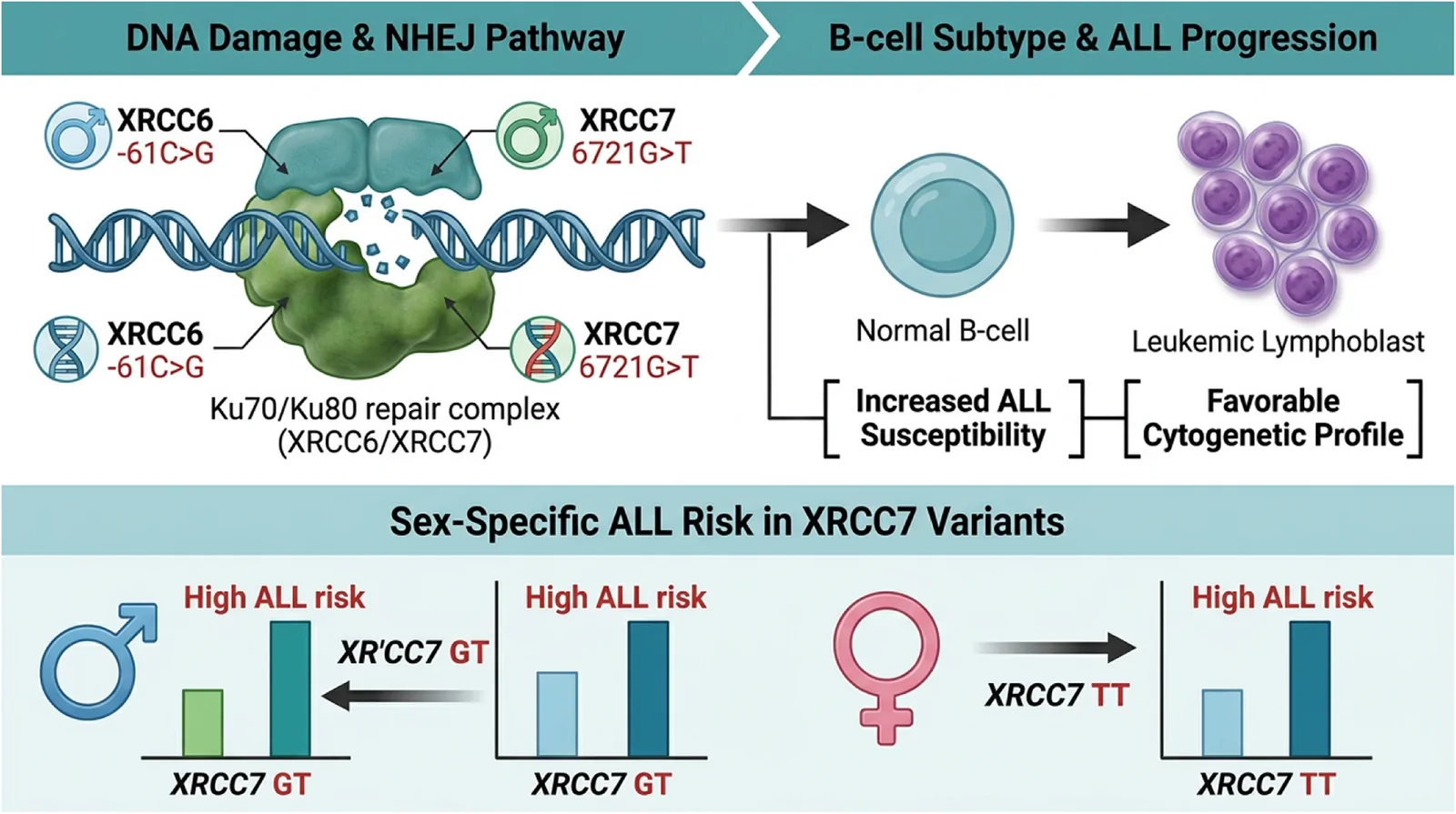

**Supplementary Information:**

The online version contains supplementary material available at https://doi.org/10.1186/s43046-026-00402-6.

## Introduction

Cancer is now recognized as the second largest cause of death globally, driving extensive research efforts to address the growing global burden of this disease [1, 2]. Among various types of cancer, Acute lymphoblastic leukemia (ALL) is the most prevalent type of cancer in children, representing almost one quarter of all pediatric malignancies [3].

ALL is diagnosed in approximately 3,000 to 4,000 individuals annually in the United States, with children accounting for two-thirds of these cases [4]. From 2006 to 2014, the average number of new cases of ALL in Iranian kids under 15 years old each year was about 2.25 out of every 100,000 children. High-risk clusters (hotspots) for ALL were found in southern and eastern provinces, including Fars, Kohgiluyeh, and Boyer-Ahmad [5]. All lymphoid cell that is stopped at a certain stage of progress, involve immature cells with the capacity to differentiate into several lineages, can give rise to ALL [6]. The implementation of successful central nervous system (CNS)-targeted therapies, the strategic use of multi-agent systemic chemotherapy administered over an extended period (2–3 years), and notable advancements in antibiotic therapies and blood product support were all credited with the early improvements in treatment outcomes during the 1960s and 1970s [7–9].

Several genetic changes that affect lymphoid stem cells cause ALL and other malignancies by causing immature lymphocytes (blast cells) to proliferate quickly. These genetic alterations have a major impact on diagnosis, risk assessment, and focused treatment and are essential in identifying the different subtypes of ALL [10, 11]. A comprehensive physical examination, medical history, and laboratory tests such as bone marrow biopsy and genetic testing are needed to the diagnosis of ALL in order to confirm the existence of malignant cells [12].

The exact mechanisms and causes of ALL are yet unknown. Research, however, points to a tenable theory incorporating a series of crucial occurrences. Leukemogenesis is initially initiated by chromosomal translocations or fusion genes, which act as the primary hits.

Subsequently, additional genetic or epigenetic alterations, such as mutations or gene deletions, serve as second hits, cooperating to drive the progression of ALL [13, 14]. Approximately 75% of childhood ALL cases exhibit recurring chromosomal alterations, which can be detected using techniques like karyotyping, fluorescence in situ hybridization (FISH), or molecular methods [15]. (t(12;21), (t(1;19)) are examples of common chromosomal rearrangements. (Philadelphia chromosome, t(9;22)), rearrangements involving MLL at 11q23 [15, 16].

One of the key originating points is believed to stem from the incorrect repair of double-strand DNA breaks (DSBs) throughout the nonhomologous end-joining (NHEJ) process [17]. The X-ray repair cross-complementing family *XRCC6* (Ku70) and *XRCC7* (Ku80) genes are crucial components of the NHEJ pathway, one of the primary mechanisms for repairing DSBs. These breaks are among the majority harmful forms of DNA impairment, and their repair is critical for preserving genomic stability [18, 19]. The Ku70/Ku80 heterodimer binds DSBs with high affinity, acting as a scaffold to recruit DNA replication stress response such as the DNA-dependent protein kinase catalytic subunit (DNA-PKcs), *XRCC4*, and Ligase IV. This process is essential for resolving DNA damage produced by radiation treatment, chemotherapy, or endogenous stressors [20]. Given their essential role in DSB repair, *XRCC6* and *XRCC7* are potential therapeutic targets in cancer treatment. Inhibiting their activity could sensitize cancer cells to radiation or chemotherapy by impairing their ability to repair therapy-induced DSBs [21].

Investigations into the expression of the *XRCC6 -61 C > G (rs2267437)* and *XRCC7 6721G > T (rs7003908)* genes in connection with ALL have shown important correlations with the disease. Studies show that *XRCC6* gene polymorphisms, especially the rs5751129 variant, are related with a developed chance of ALL and a reduced ability to precisely repair NHEJ [22]. Additionally, a heightened risk of childhood leukemia has been linked to the *XRCC6* promoter genotype T-991 C, suggesting its possible role as a predictive marker [23]. Although not explicitly mentioned in the research, the involvement of *XRCC7* in DNA repair suggests a potential link to ALL. While most prior studies have concentrated on XRCC1, the roles of *XRCC6* and *XRCC7* in the NHEJ pathway remain underexplored in pediatric ALL.

The *XRCC7* gene is crucial for maintaining genomic stability. These variations gene, such as *XRCC7*, have been investigated in relation to the risk of childhood ALL [22].

Research suggests a wider genetic landscape influencing susceptibility because some polymorphisms in NHEJ-related genes, such as *XRCC4* and *XRCC6*, show significant associations with increased odds of developing ALL [22].

Grasping the molecular processes, particularly the functions of *XRCC6* and *XRCC7*, is crucial for creating effective therapies and enhancing patient results [24]. Examining polymorphisms in genes such as *XRCC6 -61 C > G* and *XRCC7 6721G > T* can help identify biomarkers that may inform personalized treatment strategies for cancers like ALL. Moreover, investigating these polymorphisms may provide insights into their impacts on cancer stem cells, a group of cells that a category of cells that play an essential part in both the onset and progression of cancer. This information might reveal possible therapeutic targets. In the context of childhood ALL, further research into these genes could enhance our understanding and management of the disease. Most studies on *XRCC* family polymorphisms have focused on *XRCC1* and adult solid tumors. Few studies have specifically examined the *XRCC6 -61 C > G* and *XRCC7 6721G > T* polymorphisms in the context of pediatric ALL, and to our knowledge, no previous study has stratified the analysis of these polymorphisms by B-cell versus T-cell lineage or by cytogenetic risk groups (favorable vs. poor risk). The aim of this study is to evaluate the polymorphisms *XRCC6 -61 C > G (rs2267437)* and *XRCC7 6721G > T (rs7003908)* in children with ALL. Thus, further research into these genes could enhance our understanding and management of childhood ALL.

## Materials and methods

Inclusion criteria for patients: children aged 1 to 18 years with a newly confirmed diagnosis of acute lymphoblastic leukemia based on bone marrow aspiration showing 20% or more blasts, confirmed by immunophenotyping. Exclusion criteria for patients: secondary leukemia, prior exposure to chemotherapy or radiotherapy, inherited bone marrow failure syndromes, or any concomitant malignancy. Sampling was performed using convenience sampling from consecutive eligible patients who were referred to our hematology-oncology referral center during the study period between 2023 and 2025.

Controls were age- and sex-matched healthy children recruited from the same geographic region (Fars province, Iran), with no personal or family history of any hematological malignancy. The study was conducted following approval by the Ethics Committee of FUMS (Code of Ethics: IR.FUMS.REC.1403.107.

### DNA extraction

The buffy coat from whole blood samples of patients who had bone marrow transplants was kept in the sample bank at the Shiraz Transplant Research Center. Genomic DNA was taken from the buffy coat using a QIAamp DNA mini kit (Qiagen, Germany), as instructed by the manufacturer [25, 26]. 

### Genotyping

The PCR protocol consisted of an initial cycle at 94°C for 5 minutes, followed by 30 cycles of 94°C for 30 seconds, 61.8°C for 50 seconds, and 72°C for 50 seconds, with a last step at 72°C for 10 minutes. Afterward, the PCR production was digested with 10 units of Pvu II (MBI Fermentas Inc.) and incubated at 37°C for 16 hours. The XRCC7 and XRCC6 fragments were then separated on 2% agarose gels to distinguish between the wild type and polymorphic variants. PCR-restriction fragment length polymorphism (PCR-RFLP) was performed using DNA fragments of the *XRCC6-61C > G* and *XRCC7 6721G > T*. For *XRCC6 -61C > G*: forward primer 5’-GGCGGCTGGACTACTGTGAT-3’ and reverse primer 5’-GCGCTGGGTTCTTGTTGTTG-3’ and for *XRCC7 6721G > T*: forward primer 5’-TGGCTGGTCTTCTGGGTGTT-3’ and reverse primer 5’-GCCCAGCTGCTTCTTCAACT-3. For quality control, 10% of all samples were randomly selected and re-genotyped, which showed 100% concordance. Additionally, negative controls containing no DNA template were included in every PCR run to rule out contamination. Five units of BanI (with cut site: 5′-GGCGCC) and Pvu II (with cut site: 5′-CAGCTG) were used to digest about 5 µL (~ 0.1 µg) of the *XRCC6-61 C > G* and *XRCC7 6721G > T* amplified products, separately.

### Statistical analysis

Allele and genotype frequencies were determined for both patients and controls using direct gene counting. Arlequin V311 and the Statistical Package for the Social Sciences (SPSS) version 20 were used for the analysis. The Chi-square test, Fisher’s exact test, and Armitage test were used to compare the allele/genotype frequencies between the two groups. For relative risks, odds ratios and 95% confidence intervals (CIs) were computed. All reported p values were two-tailed, and a probability value of *P* < 0.05 was deemed statistically significant.

Sample size was calculated using the standard formula for case-control studies. We assumed a minor allele frequency of 0.25, a statistical power of 80%, an alpha level of 0.05, and an expected odds ratio of 2.0 based on previous similar studies. The minimum required sample size was calculated as 85 individuals per group. To account for potential dropouts or incomplete data, we enrolled 100 patients and 100 controls. Post-hoc power calculation showed greater than 80% power for our main associations. Hardy–Weinberg equilibrium (HWE) was assessed for the control group using the chi-square goodness-of-fit test. To account for multiple comparisons, we applied. Bonferroni correction was applied only to the immunophenotype and cytogenetic subgroup analyses, which comprised 5 independent tests (B-cell vs. T-cell, and 4 cytogenetic risk comparisons: favorable vs. poor risk for genotype and allele frequencies). For sex-based comparisons (Table 3), no correction was applied, and P-values are presented as nominal. These findings should be considered exploratory and hypothesis-generating.

## Results

In this case-control study, 100 children with ALL were recruited from our referral hospital specializing in hematological cancers between 2023 and 2025. The clinical and laboratory characteristics of the ALL patients are detailed in Table 1. An oncologist confirmed the ALL diagnosis through morphological examination, cytochemistry, and immunophenotyping. Additionally, we gathered laboratory and clinical data, such as hemoglobin (Hb) levels, blast percentage, total blood count, and French-American-British (FAB) classification. Genotype distributions in the control group did not deviate from Hardy–Weinberg equilibrium for either polymorphism (*P* > 0.05). Both polymorphisms were in HWE in the control population (XRCC6 -61 C > G: *P* = 0.31; XRCC7 6721G > T: *P* = 0.42). The study was conducted following informed consent obtained from patients or their legal parents. Also, Researchers have received a specific grant from the Fasa University of Medical Science.

**Table 1:** Patient characteristics

Patient characteristics	No. of patients (%)
Sex	
Male	67(67.0)
Female	33(33.0)
Median age (years)	12(range 5–18)
Blood count	
WBC (× 106)	36.2(range 0.6–290)
HB (g/dl)	7.1(range 2.9–15.1)
PLT (× 106)	53 (range 4-283)
Smear (% blasts)	
PB	58 (range 19–90)
BM	69(range 20–96)
Cytogenetic/molecular risk	
Favorable risk	86(86%)
Poor risk	14(14.0%)

*Abbreviations*: *ALL* acute lymphoblastic leukemia, *WBC* white blood cell count, *HB* hemoglobin, *PLT* platelet count, *PB* peripheral blood, *BM* bone marrow

### Genetic polymorphism of XRCC6 -61 C > G, XRCC7 6721G > T and ALL

In this investigation were discovered significant correlations between the increased prevalence of the CC genotype and the CG genotype of *XRCC6-61 C > G* with ALL patients (*P* = 0.006; OR: 2.1, 95%CI: 1.1–3.9; *P* = 0.01; OR: 0.61, 95%CI: 0.3–1.2; respectively). However, after Bonferroni correction (adjusted *P* < 0.01), these associations were considered nominal and should be interpreted with caution. Also, in both male and female ALL patients, significant correlations between the GT and TT genotypes of *XRCC7 6721G > T* were discovered (*P* = 0.02; OR: 0.38, 95%CI: 0.14–1.02; *P* = 0.05; OR: 6.2, 95%CI: 0.83–2.7; respectively). The association for the TT genotype in females (*P* = 0.05) is considered nominal after Bonferroni correction and requires validation in larger cohorts (Tables 2, 3 and 4).

**Table 2:** Genetic polymorphism of XRCC6 -61 C > G, XRCC7 6721G > T and children with acute lymphoblastic leukemia

Gene	Genotype	Patients (ALL) . No. (%)	Controls. No. (%)	*P* value	OR	%95Cl
XRCC6	CC	64 (64.0)	45 (45.0)	**0.006**	2.1	1.1_3.9
	GC	20 (20.0)	29 (29.0)	**0.01**	0.61	0.3_1.2
	GG	16 (16.0)	26 (26.0)	0.08	0.54	0.25_1.1
	C allele	148 (74.0)	119 (59.5)	**0.002**	1.9	1.2_3.03
	G allele	52 (26.0)	81 (40.0)			
XRCC7	GT	55 (55.0)	49 (49.0)	0.31	1.2	0.7_2.2
	GG	27 (27.0)	29 (29.0)	0.31	0.9	0.46_1.7
	TT	18 (18.0)	22 (22.0)	1.5	0.7	0.36_1.6
	G allele	109 (54.5)	107 (53.5)	0.84	1.04	0.6_1.5
	T allele	91 (45.0)	93 (46.5)			

Statistical tests: Chi-square test was used for comparing genotype and allele frequencies between male and female ALL patients. *P*-values were two-tailed, and *P* < 0.05 was considered statistically significant. Reference categories: For XRCC6, GG genotype and G allele were used as references; for XRCC7, GG genotype and G allele were used as references. Bold *P*-values indicate statistical significance (*P* < 0.05)

*Abbreviations*: *ALL* acute lymphoblastic leukemia, *CI* confidence interval, *OR* odds ratio

**Table 3:** Genetic polymorphism of XRCC6 -61 C > G, XRCC7 6721G > T and gender in children with acute lymphoblastic leukemia

Gene	Genotype	Male (ALL) . No. (%)	Female. No. (%)	*P* value	OR	%95Cl
XRCC6	CC	33 (49.2)	18 (54.5)	0.61	0.8	0.32_2.02
	GC	24 (35.8)	12 (36.3)	0.95	0.97	0.37_2.5
	GG	10 (14.9)	3 (9.09)	0.41	1.7	0.47_10.6
	C allele	90 (67.1)	48 (72.7)	0.42	0.76	0.37_1.5
	G allele	44 (32.8)	18 (27.2)			
XRCC7	GT	27 (40.2)	21 (63.6)	**0.02**	0.38	0.14_1.02
	GG	29 (43.2)	11 (33.3)	0.33	1.5	0.59_4.06
	TT	11 (16.4)	1 (3.03)	0.05	6.2	0.83_2.7
	G allele	85 (63.4)	43 (65.1)	0.81	0.92	0.47_1.7
	T allele	49 (36.5)	23 (34.8)			

Statistical tests: Chi-square test was used for comparing genotype and allele frequencies between male and female ALL patients. *P*-values were two-tailed, and *P* < 0.05 was considered statistically significant. Reference categories: For XRCC6, GG genotype and G allele were used as references; for XRCC7, GG genotype and G allele were used as references. Bold *P*-values indicate statistical significance (P < 0.05)

*Abbreviations*: *ALL* acute lymphoblastic leukemia, *CI* confidence interval, *OR* odds ratio

**Table 4:** Genetic polymorphism of XRCC6 -61 C > G, XRCC7 6721G > T and relapse in children with acute lymphoblastic leukemia

Gene	Genotype	Relapse (ALL) . No. (%)	No Relapsing. (%)	*P* value	OR	%95Cl
XRCC6	CC	11 (64.7)	56 (67.4)	0.82	0.88	0.26_3.2
	GC	5 (29.4)	19 (22.8)	0.56	1.4	0.34_4.9
	GG	1 (5.8)	8 (9.6)	0.62	0.58	0.01_4.9
	C allele	27 (79.4)	131 (78.9)	0.94	0.41	0.39_3.4
	G allele	7 (20.5)	35 (21.0)			
XRCC7	GT	9 (52.9)	49 (59.03)	0.64	0.7	0.24_2.5
	GG	5 (29.4)	23 (27.7)	0.88	1.08	0.26_3.7
	TT	3 (17.6)	11 (13.2)	0.63	1.4	0.22_6.2
	G allele	19 (55.8)	95 (57.2)	0.88	0.94	0.42_2.1
	T allele	15 (44.1)	71 (42.7)			

Statistical tests: Chi-square test or Fisher’s exact test was used for comparing genotype and allele frequencies between relapsed and non-relapsed ALL patients. *P*-values were two-tailed, and *P* < 0.05 was considered statistically significant. Reference categories: For XRCC6, GG genotype and G allele were used as references; for XRCC7, GG genotype and G allele were used as references. *P*-values indicate statistical significance (*P* < 0.05)

*Abbreviations*: *ALL* acute lymphoblastic leukemia, *CI* confidence interval, *OR* odds ratio

### Evaluation genetic polymorphism of *XRCC6 -61 C > G*, *XRCC7 6721G > T* in terms of T-cell /B-cell in children with ALL

During this study we assessed the genetic polymorphism of *XRCC6-61 C > G*, *XRCC7 6721G > T* according to the T-Cell /B-Cell. Higher prevalence of the CC genotype, GC, and C allele of *XRCC6-61 C > G* were shown to be significantly correlated with B-cell ALL patients in the present investigation (*P* = 0.04; OR: 2.6, 95%CI: 0.86–8.1; *P* = 0.05; OR: 0.35, 95%CI: 0.1–1.2; *P* = 0.05; OR: 2.08, 95%CI: 0.87–4.7 respectively). After Bonferroni correction (adjusted *P* < 0.01), the associations for GC genotype and C allele (*P* = 0.05) are considered nominal and should be interpreted as hypothesis-generating. Additionally, this study demonstrated a significant association between the TT genotype of *XRCC7 6721G > T* and the higher prevalence of GT genotype in B-cell ALL patients (*P* = 0.003; OR: 4.2, 95%CI: 1.3–13.2; *P* = 0.05; OR: 0.32, 95%CI: 0.08–1.2 respectively). The association for the TT genotype (*P* = 0.05) is considered nominal and requires confirmation in independent studies (Table 5).

**Table 5:** Genetic polymorphism of XRCC6 -61 C > G, XRCC7 6721G > T and Immunophenotypic Subtype (B-cell vs. T-cell) in Children with Acute Lymphoblastic leukemia

Gene	Genotype	B-Cell (ALL) . No. (%)	T-Cell. No. (%)	*P* value	OR	%95Cl
XRCC6	CC	59 (74.6)	11 (52.3)	0.04	2.6	0.86_8.1
	GC	12 (15.1)	7 (33.3)	0.05	0.35	0.1_1.2
	GG	8 (10.1)	3 (14.2)	0.58	0.67	0.14_4.3
	C allele	130 (82.2)	29 (69.04)	0.05	2.08	0.87_4.7
	G allele	28 (17.7)	13 (30.9)			
XRCC7	GT	57 (72.1)	8 (38.09)	0.003	4.2	1.3_13.2
	GG	13 (16.4)	7 (33.3)	0.08	0.39	0.1_1.3
	TT	9 (11.3)	6 (28.5)	0.05	0.32	0.08_1.2
	G allele	83 (52.5)	22 (52.3)	0.9	1.06	0.47_2.1
	T allele	75 (47.4)	20 (47.6)			

Statistical tests: Chi-square test or Fisher's exact test was used for comparing genotype and allele frequencies between B-cell ALL and T-cell ALL patients. *P*-values were two-tailed. After Bonferroni correction for 5 independent tests, the adjusted significance threshold was *P* < 0.01. Results with P between 0.01 and 0.05 are considered nominal associations. Reference categories: For XRCC6, GG genotype and G allele were used as references; for XRCC7, GG genotype and G allele were used as references. Bold *P*-values indicate statistical significance (*P* < 0.05)

*Abbreviations*: *ALL* acute lymphoblastic leukemia, *CI* confidence interval, *OR* odds ratio

### Genetic polymorphism of *XRCC6 -61 C > G*, *XRCC7 6721G > T* according to cytogenetic abnormalities status groups

In current research, we evaluated the genetic polymorphism of *XRCC6-61 C > G*, *XRCC7 6721G > T* according to the Cytogenetic abnormalities status groups. Significant correlations between a higher prevalence of the CC genotype and the C allele of *XRCC6-61 C > G* in ALL patients were discovered in the present investigation (Favorable) with regard to the status of cytogenetic abnormalities (*P* = 0.04; OR: 0.3, 95%CI: 0.81–11.5; *P* = 0.03; OR: 2.5, 95%CI: 0.87–4.7; respectively). The association for the C allele (*P* = 0.03) is considered nominal after Bonferroni correction and should be interpreted cautiously (Table 6).

**Table 6:** Genetic polymorphism of XRCC6 -61 C > G, XRCC7 6721G > T and cytogenetic/molecular risk (Favorable/poor) in children with acute lymphoblastic leukemia

Gene	Genotype	Cytogenetic/Molecular Risk.Favorable. No. (%)	Cytogenetic/Molecular Risk. Poor. No. (%)	*P* value	OR	%95Cl
XRCC6	CC	65 (75.5)	7 (50.0)	0.04	0.3	0.81_11.5
	GC	15 (17.4)	5 (35.7)	0.11	0.38	0.1_1.2
	GG	6 (6.9)	2 (14.2)	0.37	0.46	0.14_4.3
	C allele	145 (84.3)	19 (67.8)	0.03	2.5	0.87_4.7
	G allele	27 (15.6)	9 (32.1)			
XRCC7	GT	44 (51.1)	9 (64.2)	0.36	0.58	0.14_2.1
	GG	23 (26.7)	4 (28.5)	0.88	0.91	0.23_4.3
	TT	19 (22.9)	1 (7.1)	0.19	0.68	0.48_1.6
	G allele	90 (52.3)	17 (60.7)	0.4	0.71	0.28_1.7
	T allele	82 (47.6)	11 (39.2)			

Statistical tests: Chi-square test or Fisher’s exact test was used for comparing genotype and allele frequencies between favorable cytogenetic risk and poor cytogenetic risk groups. *P*-values were two-tailed, and *P* < 0.05 was considered statistically significant. Reference categories: For XRCC6, GG genotype and G allele were used as references; for XRCC7, GG genotype and G allele were used as references. Comparisons were made between favorable cytogenetic risk and poor cytogenetic risk groups. Bold *P*-values indicate statistical significance (*P* < 0.05)

*Abbreviations*: *ALL* acute lymphoblastic leukemia, *CI* confidence interval, *OR* odds ratio

Genetic model analysis showed that the dominant model for XRCC6 -61 C > G (CC vs. CG + GG) was significantly associated with ALL risk (*P* = 0.004, OR = 2.3, 95% CI: 1.3–4.1), while the recessive model did not reach significance.

## Discussion

The development of ALL is a multifactorial process influenced by numerous genes [27]. Polymorphisms within DNA repair genes may change a vulnerability of person to ALL, while mutations resulting in deficiencies in DNA repair enzymes may impede repair mechanisms [28, 29]. An essential part of the NHEJ pathway, the *XRCC6 -61 C > G* and *XRCC7 6721G > T* genes have been studied for their role in DNA repair and its relationship to ALL [30]. It is important to interpret our borderline findings and those with wide confidence intervals with caution, as they indicate statistical imprecision and require validation in larger independent cohorts.

It is important to interpret our borderline findings and those with wide confidence intervals with caution, as they indicate statistical imprecision and require validation in larger independent cohorts.

Our findings differ from those of Aref et al., who reported a protective effect of certain XRCC6 genotypes in acute myeloid leukemia (AML). however, the association for the XRCC6 CG genotype (*P* = 0.01) in our study is considered nominal after Bonferroni correction and should be interpreted with caution. This discrepancy may be explained by differences in cancer type (ALL versus AML), differences in the cell of origin (lymphoid versus myeloid lineage), and differences in ethnic and genetic backgrounds between the studied populations. Rather than the elevated risk shown in our ALL sample, Aref et al., discovered that CG/GG genotypes were associated with a lower incidence of AML compared to controls, indicating a potential protective effect in AML [31]. Similarly, Akgun et al. found that the same genotypes were correlated with a lower incidence of papillary thyroid carcinoma [32]. while Jia et al. found that the dominant model (CG/GG) and the *XRCC6 rs2267437* GG genotype were associated with an increased cancer risk across multiple malignancies [33]. These differences highlight how *XRCC6* polymorphism effects are particular to tissues and diseases. Moreover, a recent study in pediatric ALL reported that the C allele of *XRCC6* rs5751129, functionally related to the − 61 C > G site, was significantly associated with increased ALL susceptibility, accompanied by decreased *XRCC6* transcription and impaired NHEJ repair capacity. This outcome is in accordance with our research and supports the hypothesis that leukemogenesis may be influenced by reduced NHEJ efficiency or *XRCC6* expression [22]. Thus, in a Taiwanese case-control study, the TC genotype of *XRCC6 T-991 C* was found to contribute to a higher risk of ALL compared to the TT genotype [19].

The XRCC6 -61 C > G polymorphism is located in the promoter region and may affect the binding affinity of transcription factors, particularly Sp1, thereby altering XRCC6 transcriptional activity. The XRCC7 6721G > T polymorphism is located in intron 8 and may affect mRNA splicing efficiency or transcript stability. Both mechanisms can reduce Ku70/Ku80 heterodimer formation, impair double-strand break recognition, delay NHEJ, and lead to accumulation of chromosomal translocations such as t(12;21) and t(9;22), which are commonly observed in pediatric ALL. However, a Chinese study revealed that *XRCC6-61 C > G* CG/GG genotypes were related to a decreased incidence of AML, highlighting the ethnic and cancer-type specificity of *XRCC6* variant effects [31]. All of these results support the idea that decreased XRCC6 expression or promoter activity brought on by certain polymorphisms may hinder NHEJ-mediated DNA repair, causing genomic instability and mutation accumulation in hematopoietic cells, which in turn drives ALL pathogenesis [34].

The *XRCC7 6721G > T* variant, located within intron 8, has been implicated in altered transcript stability and in disturbances of mRNA splicing, both of which can affect the efficiency of DNA double-strand break repair. Earlier investigations have largely focused on this polymorphism in solid tumors including glioma, bladder, and cervical cancers as well as in disorders such as systemic lupus erythematosus (SLE) and male infertility [34–39]. According to experimental findings, the T allele has been associated to decreased NHEJ repair activity and *XRCC7* mRNA levels, which may increase genomic instability [34, 35, 40]. In contrast to these prior observations, our findings introduce new evidence for a sex-dependent relationship between the *XRCC7 6721G > T* variant and ALL. In our data set, the GT genotype appeared to increase susceptibility among males, whereas the TT genotype showed a stronger association in females [22]. Nevertheless, the association for the TT genotype in females (*P* = 0.05) is nominal and requires validation in larger independent cohorts due to the small subgroup sample size. This pattern suggests that functional variation in NHEJ-related genes may influence leukemogenesis differently in men and women. Consistent with this concept, prior investigations have indicated that the GT and TT genotypes are reported that a greater incidence of glioma, particularly among middle-aged men [41]. The *XRCC7 TT* genotype (rs7003908) was associated with an increased risk of ALL after controlling for parental tobacco smoking, according to a case-control study of 200 healthy controls and 99 ALL patients. Notably, when smoking history was omitted from the analysis, the GT and TT genotypes alone did not significantly increase risk, pointing to a gene–environment interaction in leukemogenesis [42]. Mechanistically, chromosomal rearrangements and translocations molecular characteristics of infant and pediatric ALL may be facilitated by the genotype of TT decreased *XRCC7* expression and decreased NHEJ fidelity [34, 40].

In our analysis, the distribution of genotypes related to the *XRCC6 − 61 C > G* variant showed a clear shift among individuals diagnosed with B-cell ALL. Both the CC and GC genotypes, along with the C allele, appeared more frequently in the patient group than in controls. However, the associations for the GC genotype (*P* = 0.05) and the C allele (*P* = 0.05) in B-cell ALL are nominal and should be considered hypothesis-generating rather than definitive. This trend echoes the findings of Jia et al., who suggested that the C allele of *XRCC6 rs5751129* may heighten vulnerability to several malignancies, indicating that this variant could influence cancer risk across diverse tissues [33]. Akgun et al. also reported an interesting pattern in thyroid cancer, where the C allele was linked to increased *XRCC6* expression, while the G allele seemed to exert a modest protective effect [32]. Most prior investigations, however, have focused largely on solid tumors. By contrast, our study provides the first indication that the C allele may increase susceptibility specifically within the B-cell lineage, hinting at a lineage-restricted genetic effect in hematologic cancers. A comparable pattern emerged for the *XRCC7 6721G > T* polymorphism. We observed that the GT and TT genotypes occurred more often among patients with B-cell ALL, suggesting that this variant may contribute to disease development. The association for the TT genotype in B-cell ALL (*P* = 0.05) is nominal and should be interpreted with caution until replicated in larger studies. Similar to our findings about the higher prevalence of the TT genotype among B-cell ALL patients, Farokhian et al. also found that the TT genotype (*XRCC7 G6721T)* enhanced the risk of developing ALL in a juvenile cohort [42]. However, unlike our investigation, their analysis did not stratify individuals based on B-cell or T-cell lineage, reducing the specificity of their genotype-disease correlation.

In our analysis, the CC genotype and the C allele of the *XRCC6 − 61 C > G* polymorphism was found more frequently among ALL patients who carried favorable cytogenetic abnormalities. The association for the C allele (*P* = 0.03) is considered nominal after Bonferroni correction and requires confirmation in independent cohorts. This pattern suggests that the C allele may play a context-specific, possibly protective role in leukemogenesis. Although the strength and direction of these effects seem to vary across tumor types and populations, previous research by Jia et al. suggested that *XRCC6* polymorphisms close to regulatory or promoter regions can affect cancer susceptibility via changing DNA repair efficiency [33, 43]. It is important to acknowledge that not all studies have reported positive associations for these polymorphisms. Pei et al. found no significant association between XRCC6 promoter genotype T-991 C and ALL risk in their Taiwanese pediatric cohort, suggesting that the impact of *XRCC6* variants may differ across populations [23]. Similarly, Chang et al. investigated multiple NHEJ pathway genes including *XRCC6* and *XRCC7* in a large North American cohort and did not observe significant main effects on ALL susceptibility [44], although they noted potential gene-environment interactions. Furthermore, Farokhian et al. reported that while the *XRCC7 TT* genotype was associated with ALL risk when stratified by parental smoking, the genotype alone (without consideration of environmental exposure) did not show a significant overall association [42]. These discrepant findings highlight the possibility that the effects of *XRCC6* and *XRCC7* polymorphisms on ALL susceptibility may be modified by ethnic background, environmental exposures, or other genetic factors. The lack of replication in some studies underscores the need for validation in larger, well-characterized cohorts from diverse populations [22].

Evidence specifically addressing *XRCC6 − 61 C > G* in leukemia is still scarce, but studies in other malignancies indicate that the clinical impact of this polymorphism may vary depending on genetic background and environmental exposures. In support of this theory, Farokhian et al. found a significant interaction with parental smoking but no overall association between XRCC6 variants and ALL in Iranian children, highlighting the possibility that environmental factors could alter the contribution of DNA-repair genes to leukemia risk [42]. *XRCC6 − 61 C > G* and *XRCC7 6721G > T* polymorphisms have not yet been studied in relation to cytogenetic subgroups of ALL. Therefore, our discovery that patients with favorable-risk cytogenetics seem to have a higher frequency of the CC genotype may be unique. When considered collectively, these findings highlight the need for validation in larger, ethnically diverse populations and imply that *XRCC6*-related genetic effects on ALL etiology may rely on cytogenetic context [33, 42, 45].

Several limitations warrant consideration. First, our study population was drawn exclusively from Fars province, Iran, which may limit generalizability. Additionally, all associations with P-values between 0.01 and 0.05 (including XRCC6 CG, XRCC7 TT in females, XRCC6 GC and C allele in B-cell ALL, XRCC7 TT in B-cell ALL, and XRCC6 C allele in favorable cytogenetics) are considered nominal and hypothesis-generating. These findings should not be over interpreted and require validation in larger, well-powered studies. Although the minor allele frequencies observed in our control group (*XRCC6 -61G*: 0.38; *XRCC7 6721T*: 0.47) were consistent with gnomAD data for Middle Eastern populations (*XRCC6*: 0.35–0.40; *XRCC7*: 0.42–0.48), population stratification cannot be completely excluded. Future studies should include ancestry-informative markers in diverse populations. Second, the wide confidence intervals observed for some associations (*XRCC7 TT* genotype in females: OR = 6.2, 95% CI: 0.83–2.7) reflect small subgroup sample sizes and limit the precision of our estimates. Consequently, our subgroup findings should be considered exploratory and hypothesis-generating, requiring validation in larger independent cohorts. Another significant limitation is that we did not investigate gene-environment interactions, which are particularly relevant for DNA repair genes. Farokhian et al. demonstrated a significant interaction between *XRCC7* variants and parental tobacco smoking in ALL susceptibility, with the TT genotype conferring increased risk only in the presence of smoking exposure [42]. We did not collect detailed information on environmental exposures and therefore could not assess such interactions. Future investigations should include comprehensive exposure assessment to evaluate potential gene-environment interactions and identify high-risk subgroups.

## Supplementary Information

Supplementary Material 1.

## Data Availability

No datasets were generated or analysed during the current study.
