# Supplementary material for: Association of XRCC6 -61 C > G and XRCC7 6721G > T polymorphisms with susceptibility to pediatric acute lymphoblastic leukemia
Source: J Egypt Natl Canc Inst. 2026 Oct 16;38:78. doi: 10.1186/s43046-026-00402-6 (PMC13675453; doi:10.1186/s43046-026-00402-6)
Supplement: Supplementary file 1 [file 43046_2026_402_MOESM1_ESM.docx]

**Supplementary Table S1: Genetic Inheritance Models for XRCC6 -61C>G and XRCC7 6721G>T Polymorphisms in Association with Pediatric ALL**

| Gene | Model | Genotype Comparison | Cases n (%) | Controls n (%) | OR (95% CI) | P-value | P-adjusted* |
| --- | --- | --- | --- | --- | --- | --- | --- |
| *XRCC6*  -61C>G (rs2267437) | Dominant | CC vs. CG+GG | 64 (64.0) | 45 (45.0) | 2.17 (1.22–3.87) | **0.008** | 0.02 |
|  | Recessive | GG vs. CC+CG | 16 (16.0) | 26 (26.0) | 0.54 (0.27–1.09) | 0.08 | 0.16 |
|  | Additive | C allele vs.  G allele | 148 (74.0) | 119 (59.5) | 1.93 (1.23–3.03) | **0.004** | **0.008** |
| *XRCC7*  6721G>T (rs7003908) | Dominant | GT+TT vs. GG | 73 (73.0) | 71 (71.0) | 1.10 (0.60–2.02) | 0.75 | 1.00 |
|  | Recessive | TT vs. GT+GG | 18 (18.0) | 22 (22.0) | 0.78 (0.39–1.55) | 0.47 | 0.94 |

**Supplementary Materials**

Supplementary Methods: Power Calculations

Post-hoc power calculations were performed using the method described by Dupont and Plummer (1990) for unmatched case-control studies. Assuming a two-sided alpha level of 0.05, a minor allele frequency of 0.25 in controls, and an odds ratio of 2.0, the study had 82% power to detect a significant association with a sample size of 100 cases and 100 controls. For the subgroup analyses (B-cell ALL, n = 79; favorable cytogenetics, n = 86), the power was reduced (range: 45–70%) for detecting similar effect sizes, indicating that the null findings in some subgroups may be due to limited statistical power rather than true absence of association.
